# Supplementary material for: Homeostatic synaptic plasticity rescues neural coding reliability
Source: Nat Commun. 2023 May 24;14:2993. doi: 10.1038/s41467-023-38575-6 (PMC10209050; doi:10.1038/s41467-023-38575-6)
Supplement: Supplementary file 1 — Supplementary Information [file 41467_2023_38575_MOESM1_ESM.pdf]

Supplementary information

Homeostatic Synaptic Plasticity Rescues Neural Coding Reliability

Eyal Rozenfeld[1, 2, ¥], Nadine Ehmann[3, ¥], Julia E. Manoim[1], Robert J. Kittel[3, *] and Moshe Parnas[1, 2, *]

1. Department of Physiology and Pharmacology, Sackler School of Medicine, Tel Aviv University, Tel Aviv 69978, Israel
2. Sagol School of Neuroscience, Tel Aviv University, Tel Aviv 69978, Israel
3. Department of Animal Physiology, Institute of Biology, Leipzig University, 04103 Leipzig, Germany

¥ These authors contributed equally

* Co-Corresponding authors, email: mparnas@tauex.tau.ac.il, kittel@uni-leipzig.de

Contents: Supplemental Figures 1-13 and supplementary Table 1

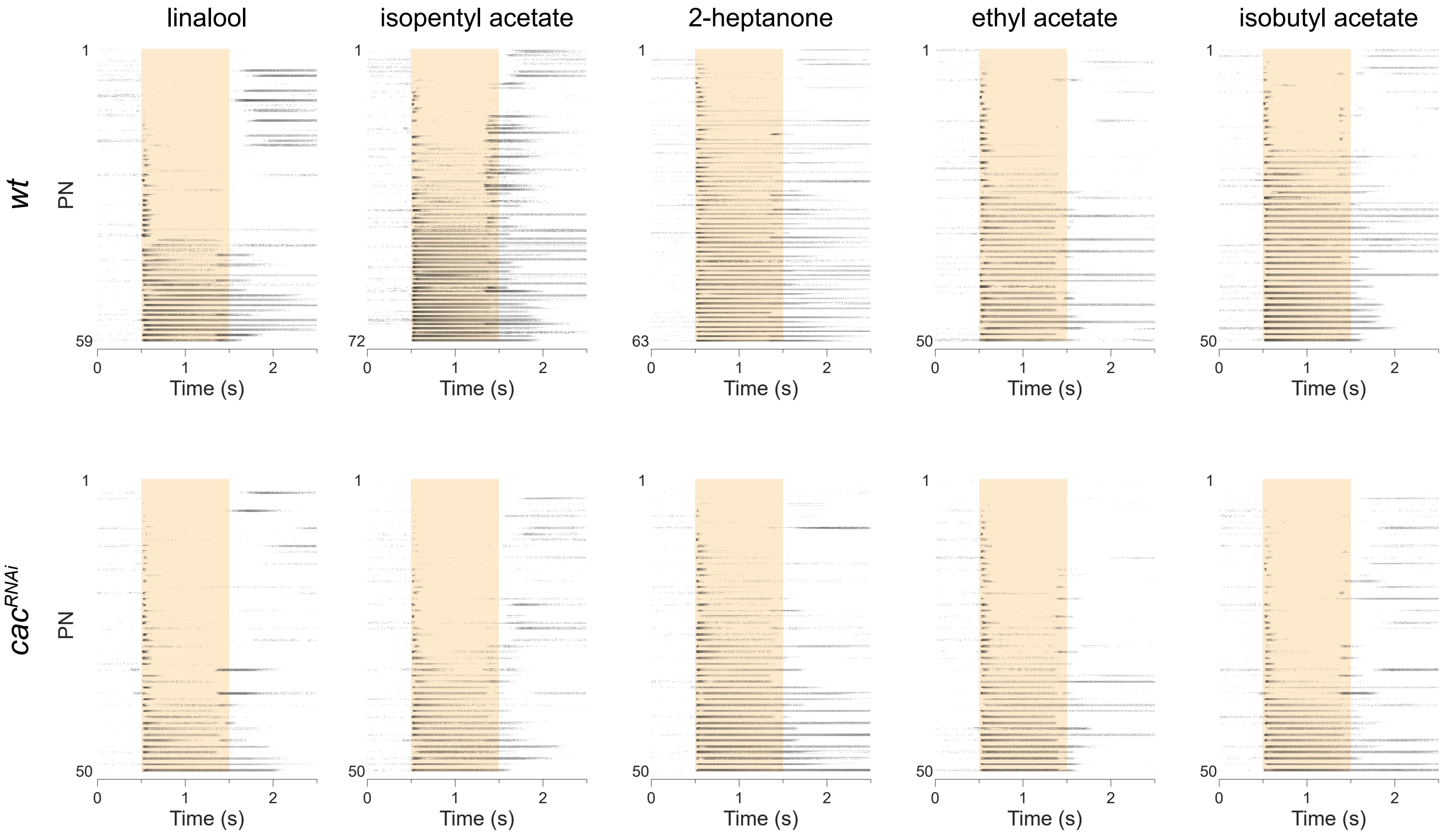

Figure S1: PN population responses to odor stimulation
Raster plots of PN population responses to five odors as indicated (final odor dilution of $5X10^{-2}$) in *wt* flies and *UAS-cac*^{RNAi} in ORNs. Each neuron was presented with 10 repetitions of the olfactory stimulus. *Orco-GAL4* drove *UAS-cac*^{RNAi} in ORNs and *GH146-QF* drove *QUAS-GFP* in PNs (n=45-72).

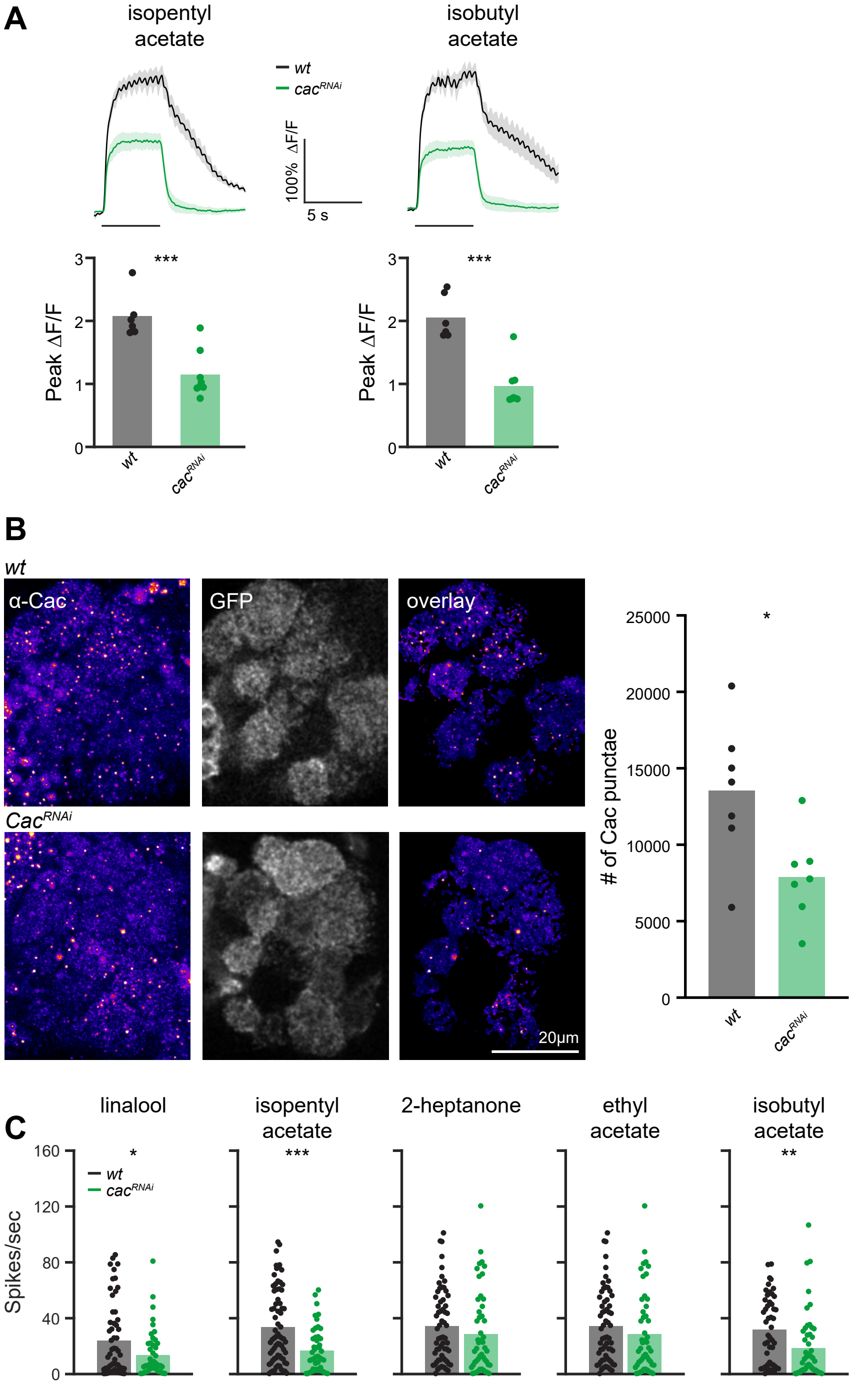

A
isopentyl acetate
isobutyl acetate
wt
cacRNAi
100% ΔF/F
5 s
Peak ΔF/F
***
wt
cacRNAi
B
wt
α-Cac
GFP
overlay
CacRNAi
20μm
# of Cac punctae
*
C
linalool
isopentyl acetate
2-heptanone
ethyl acetate
isobutyl acetate
Spikes/sec
*
***
**

Figure S2: Knockdown of *cac* reduces odor-stimulated calcium signals in ORNs and PN firing rates

A. *Top*, averaged traces ± SEM (shading) of odor responses (odor pulse is indicated with a black bar) obtained from a single plane of the entire AL for *wt* or RNAi flies. *Orco-GAL4* drove *UAS-cac^{RNAi}* along with *UAS-GCaMP6f*. *Bottom*, peak ΔF/F of odor responses for the traces presented in the *top* panel. As expected, a significant decrease in Ca^{2+} signals was observed for *cac^{RNAi}*. (*wt,* n=6; *cac^{RNAi},* n=8 flies). Two sided permutation test, $p<0.001$.

B. To validate *cac* knockdown at ORN-PN synapses, whole mounts were stained with an antibody against Cac[1] in control (upper panel; *orco-Gal4*/+; *GH-146-QF,QUAS-GFP*/+) and *cac^{RNAi}* flies (lower panel*; orco-Gal4*/*UAS-cac^{RNAi}*; *GH146-QF,QUAS-GFP*/+*)*. The analysis of Cac signals (fire) was restricted to AZs with excitatory PNs as postsynaptic partners trough an overlay with the respective mask generated by imaging *GH146* driven GFP (grey). *Right*, confocal analysis yielded a lower number of Cac punctae at ORN-PN synapses upon *cac* knock-down in ORNs, confirming the RNAi efficacy (n=7). Two sided two sample t-test, $p=0.016$.

C. Mean firing rate of PNs in response to five odors as indicated (final odor dilution of $5X10^{-2}$) for *wt* flies and *cac^{RNAi}*. Each data point shows the average of 10 responses to a 1 s odor stimulus and 1 s following the odor stimulus. *Orco-GAL4* drove *UAS-cac^{RNAi}* in ORNs and *GH146-QF* drove *QUAS-GFP*. For most odors, a significant decrease in firing rate was observed [wt, n=58 (linalool), n=49 (isobutyl acetate, ethyl acetate), n=71 (isopentyl acetate), n=62 (2-heptanone); *cac^{RNAi}*, n=49 flies]. Two sided permutation test, $p=0.014$ (linalool), $p<0.001$ (isopentyl acetate), $p=0.007$ (isobutyl acetate).

For all panels * $p<0.05$, ** $p<0.01$, *** $p<0.001$.

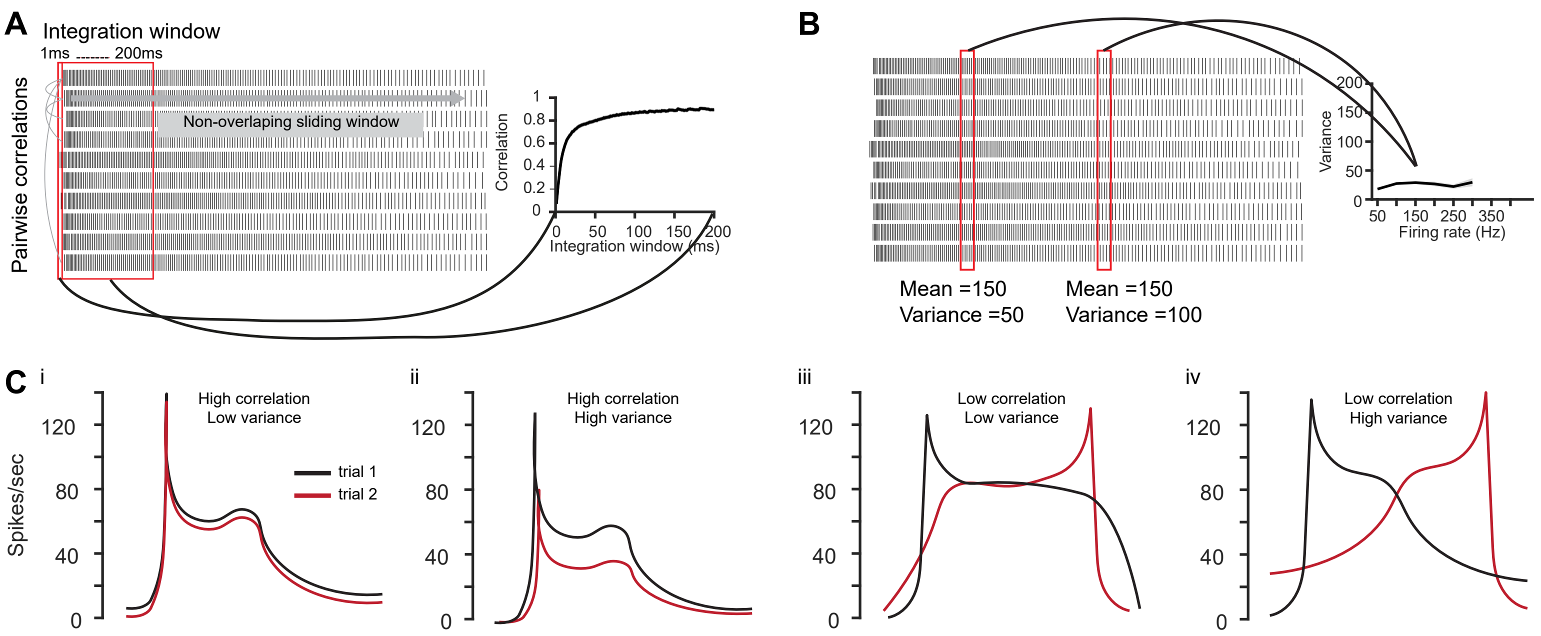

Figure S3: Graphical description of the reliability analysis

A. Temporal reliability analysis. Pairwise correlations were performed between the 10 repetitions of each odor-neuron combination. Correlations were calculated using increasing, non-overlapping integration windows from 1 to 200 ms with 1 ms intervals. The 45 pairwise correlation values for each condition (i.e. between the 10 repetitions of a given odor for a single PN) were averaged to a single correlation value for this condition. All correlation values for all PNs and odors were pooled to yield the correlation curve on the right.

B. Firing rate reliability analysis. Spike trains were binned using 20 ms windows. The firing rate and variability was calculated for each individual bin. The mean variance value for all bins having the same firing rate is presented on the variance curve on the right. Variance values are pooled across neurons, odors, and time.

C. Temporal reliability analysis and firing rate reliability analysis capture different aspects of trial-to-trial coding reliability. Correlation captures the general shape of the PSTH regardless of momentary changes in firing rate at a given point in time (i and ii). Inter-trial variability captures the momentary changes in firing rates regardless of the PSTH temporal dynamics (iii and iv).

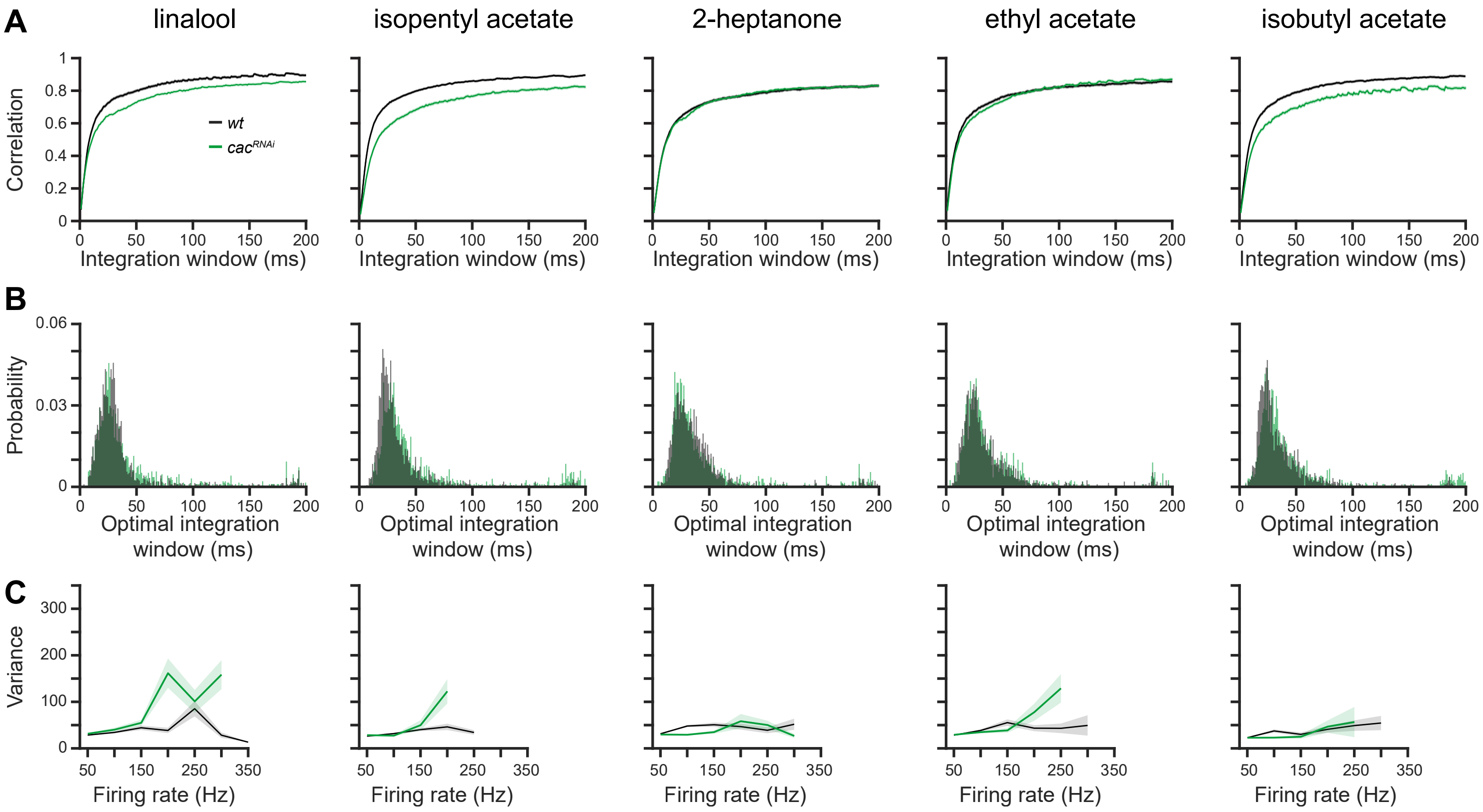

Figure S4: Single odor analyses of response reliability

A. Temporal reliability analysis as performed in Figure 1D but for single odors as indicated. Data obtained from Figure 1 and Figure S1. For all odors, a final odor dilution of $5X10^{-2}$ was used.

B. The optimal integration window analysis as performed in Figure 1E but for single odors as indicated. Data obtained from Figure 1 and Figure S1. For all odors, a final odor dilution of $5X10^{-2}$ was used.

C. Firing rate reliability analysis as performed in Figure 1F but for single odors as indicated. Data obtained from Figure 1 and Figure S1. For all odors, a final odor dilution of $5X10^{-2}$ was used. Error bands represent SEM.

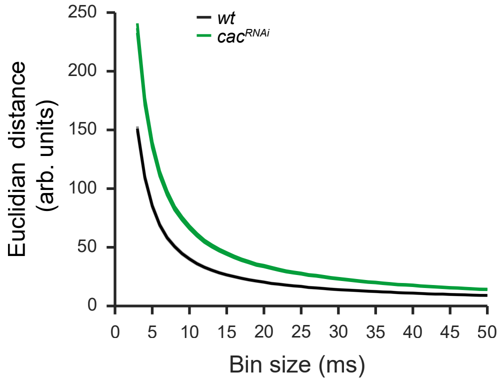

Figure S5: Reduced reliability of *cac*^{RNAi} in ORNs is also observed using Euclidean distance measurement

Pairwise Euclidean distance was calculated between the 10 repetitions of each odor-neuron combination for the data in Figure S1. Distances were calculated using increasing, nonoverlapping integration windows from 3 to 50 ms with 1 ms intervals. For each bin size, the data were normalized to the mean firing rate of the 10 repetitions of a specific odor. The 45 pairwise distances values for each condition (i.e. PN and odor) were averaged to a single distance value. All distance values for all PNs and odors were pooled.

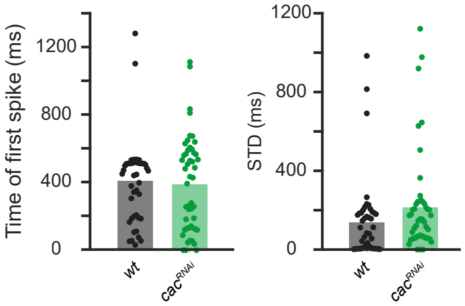

Figure S6: Controls for first spike analysis
Analysis of the latency of the first spike (left) and jitter of the first spike (right) in PNs as performed in Figure 1J, K but in response to mock odor application (i.e. no odor was actually applied) for *wt* flies or *cac*^{RNAi} in ORNs (*wt*, n=47; *cac*^{RNAi}, n= 49 flies). No change in either parameter was observed.

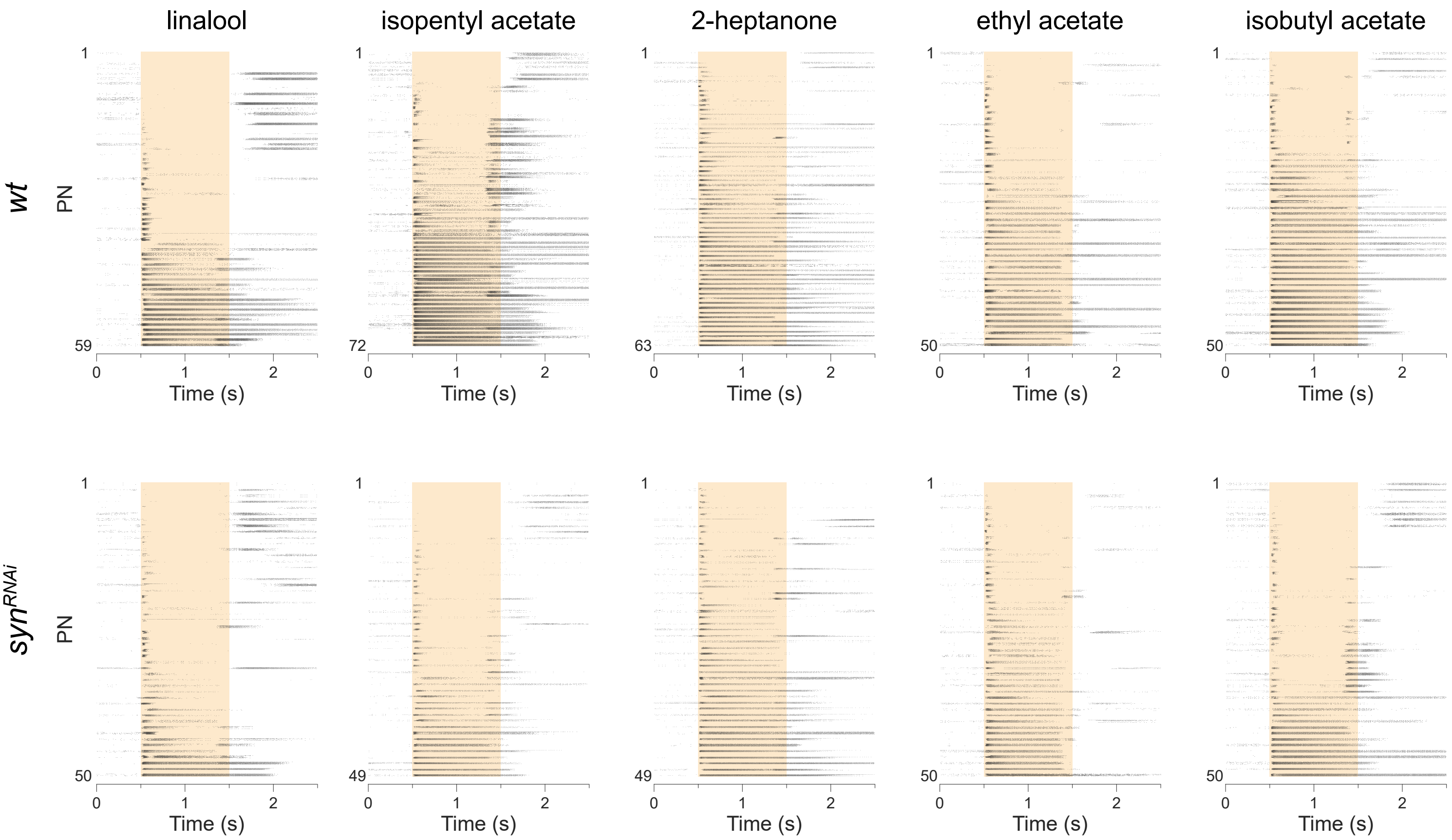

Figure S7: PN population responses to odor stimulation for Syn KD in ORNs
Raster plots of PN population responses to five odors as indicated (final odor dilution of $5X10^{-2}$) in *wt* flies and *UAS-syn^{RNAi}* in ORNs. Each neuron was presented with 10 repetitions of the olfactory stimulus. *Orco-GAL4* drove *UAS-syn^{RNAi}* in ORNs and *GH146-QF* drove *QUAS-GFP* in PNs. *wt* data is the same as in Figure S1 (n=49-72).

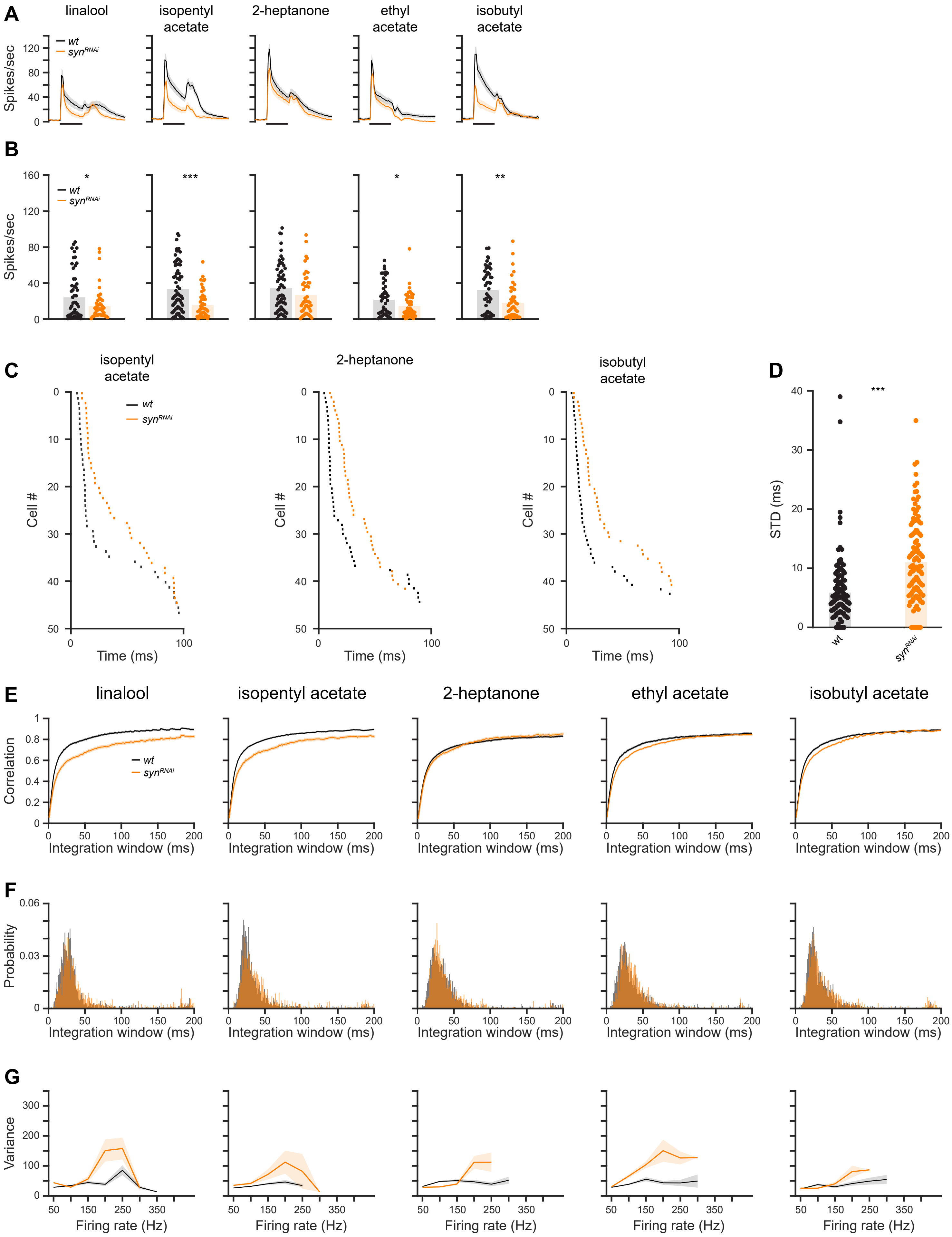

A
linalool
isopentyl acetate
2-heptanone
ethyl acetate
isobutyl acetate
Spikes/sec
wt
syn^RNAi
B
Spikes/sec
*
***
*
**
C
isopentyl acetate
2-heptanone
isobutyl acetate
Cell #
Time (ms)
D
STD (ms)
***
wt
syn^RNAi
E
linalool
isopentyl acetate
2-heptanone
ethyl acetate
isobutyl acetate
Correlation
Integration window (ms)
F
Probability
Integration window (ms)
G
Variance
Firing rate (Hz)

Figure S8: Syn KD reduce PN coding reliability

A. PSTH of PN population responses to five odors examined as indicated (shaded areas represent SEM, odor pulse is labeled with a black bar). Spike trains were binned using a 50 ms time bin. Knockdown of *syn* (orange) in ORNs resulted in decreased PN odor responses. *wt* data is the same as in Figure 1. A final odor dilution of $5X10^{-2}$ was used (n=50-72 flies). *Orco-GAL4* drove the RNAi construct and *GH146-QF* drove *QUAS-GFP*.

B. Mean firing rate of PNs in response to five odors as indicated (final odor dilution of $5X10^{-2}$) for *wt* flies and for syn^{RNAi} flies. Each data point shows the average of 10 responses to a 1 s odor stimulus and 1 s following the odor stimulus. *Orco-GAL4* drove syn^{RNAi} in ORNs and *GH146-QF* drove *QUAS-GFP*. For most odors a significant decrease in firing rate was observed. *wt* data is the same as in Figure 1. [wt, n=58 (linalool), n=49 (isobutyl acetate, ethyl acetate), n=71 (isopentyl acetate), n=62 (2-heptanone); syn^{RNAi} , n=49 (linalool, isobutyl acetate, ethyl acetate), n=48 (isopentyl acetate, 2-heptanone) flies]. Two sided permutation test, p=0.04 (linalool), p<0.001 (isopentyl acetate), p=0.026 (ethyl acetate), p=0.002 (isobutyl acetate).

C. First spike latency of PNs in response to the indicated odors for *wt* and syn^{RNAi} flies (n=48-50). Each dot represent the mean first spike latency for 10 trials of a given neuron. Data were obtained in VC configuration and PNs that did not spike within 100ms after stimulus onset were omitted. *wt* data is the same as in Figure 1.

D. First spike jitter of PN odor responses, pooled across all odors, for *wt* and syn^{RNAi} flies (*wt,* n=134; syn^{RNAi} , n=132 cells). *wt* data is the same as in Figure 1. Two sided permutation test, p<0.001.

E. Temporal reliability analysis. Pairwise correlations for each odor-neuron combination were pooled across all odors for data in Figure S7. Non-overlapping windows from 1 to 200 ms were used. syn^{RNAi} in ORNs reduces correlation values. *wt* data is the same as in Figure 1.

F. The curve saturation point (see methods) was calculated for each odor-neuron combination and pooled across all odors for data in Figure S7. syn^{RNAi} in ORNs shifts the optimal temporal integration window of PNs. *wt* data is the same as in Figure 1.

G. Firing-rate reliability analysis for data in Figure S7. Spike trains were binned using 20 ms windows. Firing rate and variability were calculated for each bin and pooled across neurons, odors, and time. Increased rate variability is observed for high firing rates. *wt* data is the same as in Figure 1. Error bands represent SEM.

For all panels * p<0.05, ** p<0.01, *** p<0.001.

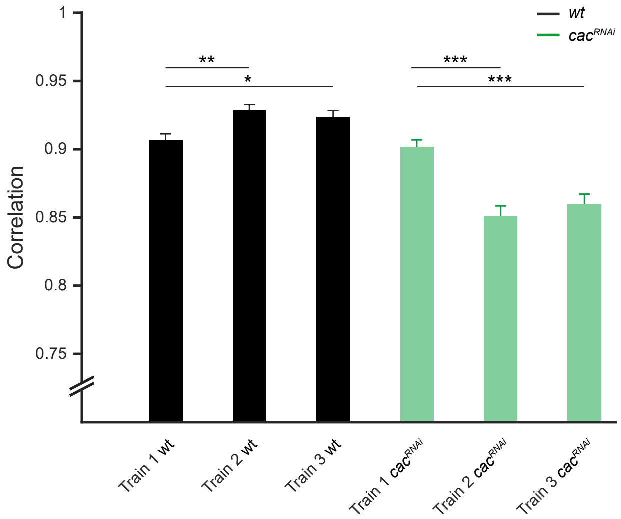

Figure S9: Cac is required for the improved correlation following repeated odor exposure
Average trial-to-trial correlations for 10 repetitions of the odor isopentyl acetate (final odor dilution of $5X10^{-2}$). Each train was separated by a 6 minutes rest interval. An increase in trial-to-trial correlations was observed for *wt* flies but not for *cac*^{RNAi} flies. Spike trains were binned using a 200 ms time window. n=20-21, error bars represents SEM. Two sided permutation test with tukey kramer correction for multiple comparisons, $p<0.001$ (*wt*), $p=0.001$ (*wt* Train 1 vs. Train 2), $p=0.02$ (*wt* Train1 vs. Train 3), $p<0.001$ (*cac*^{RNAi}), $p<0.001$ (*cac*^{RNAi} Train 1 vs. Train 2), $p<0.001$ (*cac*^{RNAi} Train 1 vs. Train 3).
For all panels * $p<0.05$, ** $p<0.01$, *** $p<0.001$.

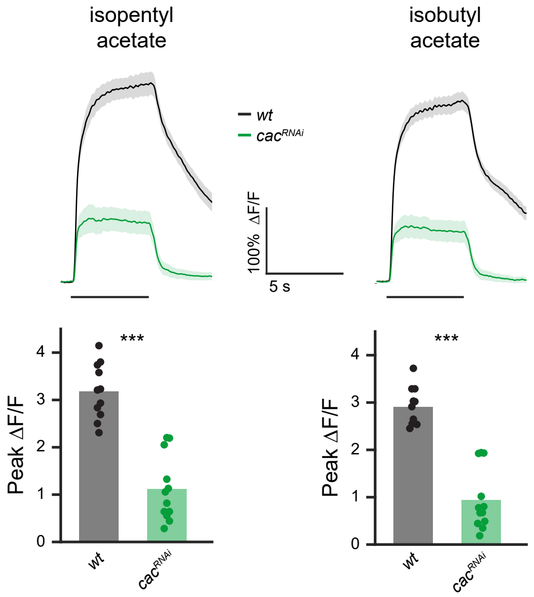

Figure S10: ORN Ca^{2+} imaging in 0 day-old flies

Top, averaged traces ± SEM (shading) of odor responses (as indicated, odor pulse is indicated with a black bar) obtained from a single plane of the entire AL for *wt* and RNAi flies. *Orco-GAL4* drove *UAS-cac*^{RNAi} along with *UAS-GCaMP6f*. *Bottom*, peak ΔF/F of odor responses for the traces presented in the top panel. A significant decrease in Ca^{2+} signals was observed for *cac*^{RNAi}. (*wt*, n=11; *cac*^{RNAi}, n=12 flies. Two sided two sample t-test and two sided permutation test, $p<0.001$. *** $p < 0.001$.

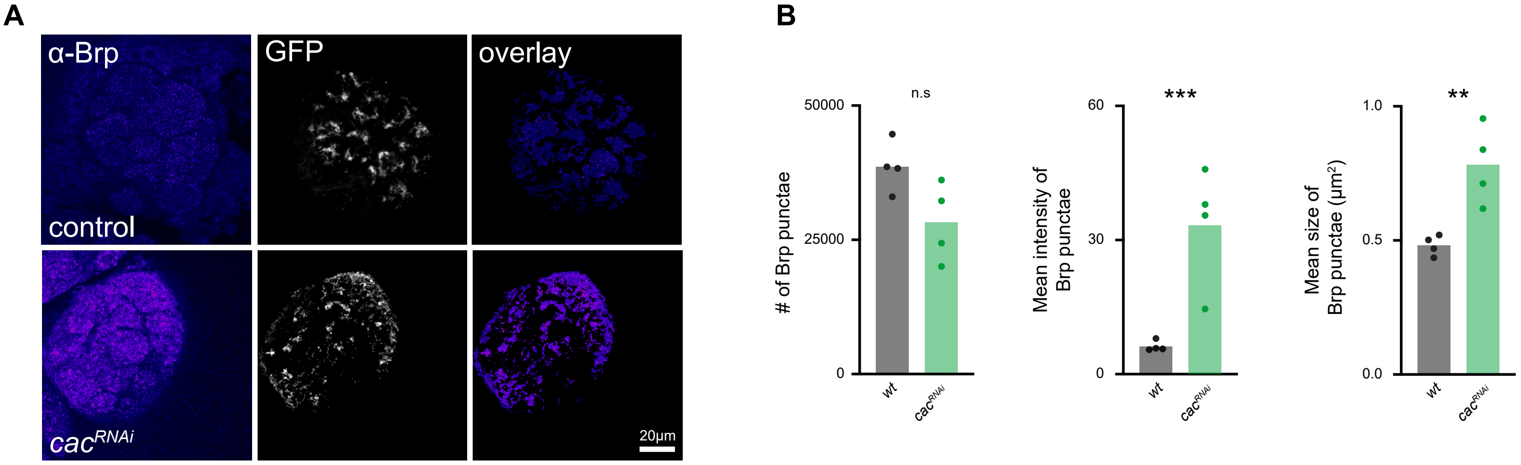

Figure S11: Structural analysis of the ORN-iLN synapse

A. Example confocal images of an individual plane through the antennal lobe in control (upper panel; *orco-Gal4/ QUAS-GFP; 449-QF/+*) and cac^{RNAi} flies (lower panel; *orco-Gal4/ QUAS-GFP; 449-QF/ UAS-*cac^{RNAi}) stained against Brp (fire). To restrict the analysis of Brp to inhibitory local synapses, the Brp signal was overlaid with masks generated from imaging endogenous GFP driven via 449-QF (GFP, grey; overlay).

B. Analysis of Brp in ORN pre-synapses shows no change in the number of Brp punctae a significant increase in the fluorescence intensity and size of Brp punctae following *cac* knockdown (*wt*, n=4; cac^{RNAi}, n=4 flies). Two sided two sample t-test and two sided permutation test, p<0.001 (Mean intensity of Brp punctae), p=0.007 (Mean size of Brp punctae).

For all panels ** p<0.01, *** p<0.001.

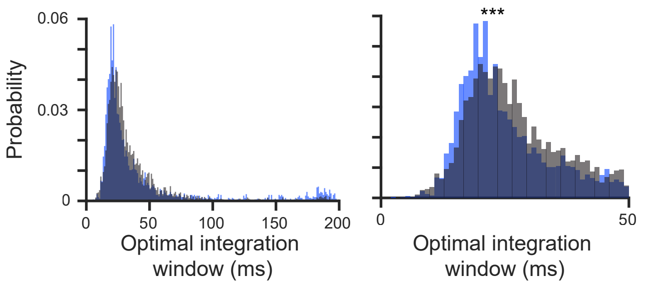

Figure S12: Knockdown of *gluClα* in PNs does not increase their integration window
Left, the curve saturation point was calculated for each odor-neuron combination and pooled across the two odors for the data in Figure 7G. *Right*, left curves are presented at a smaller scale. *gluClα* RNAi in PNs (blue) did not increase their optimal temporal integration window. If at all, it was slightly improved as reflected by a smaller integration window spread and a slight peak shift to the left. n=50-72. Two sided permutation test, $p<0.001$.
*** $p < 0.001$.

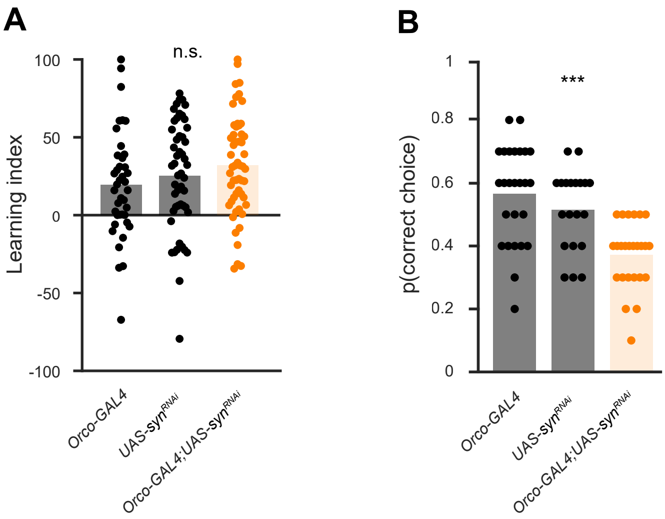

Figure S13: Knockdown of syn in ORNs reduces behavioral reliability

A. Learning performance for prolonged odor exposure at high odor concentration. No significant differences in the learning index were observed between the parental controls and syn^{RNAi} in ORNs. Each dot represents a single fly [final odor dilution of 5X10^{-2}; n=40 (*Orco-GAL4*), n=50 (*UAS- syn^{RNAi}*), n=54 (*Orco-GAL4;UAS- syn^{RNAi}*) flies]. *Orco-GAL4* data is the same as in Figure 8.

B. Behavioral reliability measure. The behavioral reliability measure was defined as the probability of correctly classifying the shock-paired odor. *syn* RNAi in ORNs reduces the behavioral reliability compared to the parental controls at high odor concentration. Each dot represents a single fly [final odor dilution of 5X10^{-2}; n=26 (*Orco-GAL4*), n=20 (*UAS-syn^{RNAi}*), n=25 (*Orco-GAL4;UAS-syn^{RNAi}*) flies]. *Orco-GAL4* data is the same as in Figure 8. Two sided permutation test, p<0.001 with tukey-kramer correction for multiple comparisons.

*** p<0.001.

Table S1: statistical analysis

Figure	data	Statistical method	comparison	P-value	Effect size	significance
Figure 1E	J-index probability *cac*^{RNAi} all odors pooled	Permutation test	1. Control (N=12133)<br>2. *cac*^{RNAi} (N=9883)	<0.001	-0.211	***
Figure 1H	Spike count IC vs. VC	Two sample paired t-test	1. IC (N=22)<br>2. VC (N=22)	0.001	-1.162	***
Figure 1I	First spike latency IC vs. VC	Two sample paired t-test	1. IC (N=22)<br>2. VC (N=22)	<0.001	1.265	***
Figure 1K	First spike jitter all odors pooled	Permutation test	1. Control (N=134)<br>2. *cac*^{RNAi} (N=127)	<0.001	-1.178	***
Figure 2C	J-index probability *cac*^{RNAi} all odors pooled	Permutation test	1. Control (N=4198)<br>2. *cac*^{RNAi} (N=4722)	<0.001	0.083	***
Figure 2F	J-index probability *cac*^{RNAi} all odors pooled	Permutation test	1. Control (N=9803)<br>2. *cac*^{RNAi} (N=7156)	<0.001	-0.253	***
Figure 3B	eEPSC amplitude (pA)	Permutation test	1. Control (N=17)<br>2. *cac*^{RNAi} (N=18)	0.789	-0.093	n.s
Figure 3D	Paired-pulse ratio (10ms interval)	Permutation test	1. Control (N=20)<br>2. *cac*^{RNAi} (N=25)	0.010	-0.638	*
	Paired-pulse ratio (30ms interval)	Permutation test	1. Control (N=21)<br>2. *cac*^{RNAi} (N=24)	0.034	-0.614	*
Figure 3E	eEPSC latency – 1Hz	Two sample t-test	1. Control (N=17)<br>2. *cac*^{RNAi} (N=18)	0.001	-1.185	**
	eEPSC latency – 10Hz	Two sample t-test	1. Control (N=17)<br>2. *cac*^{RNAi} (N=17)	<0.001	-1.402	***
	eEPSC latency – 20Hz	Two sample t-test	1. Control (N=17)<br>2. *cac*^{RNAi} (N=18)	<0.001	-1.518	***

	eEPSC latency – 60Hz	Two sample t-test	1. Control (N=17)<br>2. *cac*^{RNAi} (N=18)	<0.001	-2.053	***
Figure 3F	eEPSC jitter – 1Hz	Permutation test	1. Control (N=17)<br>2. *cac*^{RNAi} (N=18)	0.044	-0.611	*
	eEPSC jitter – 10Hz	Permutation test	1. Control (N=17)<br>2. *cac*^{RNAi} (N=17)	0.016	-0.772	*
	eEPSC jitter – 20Hz	Permutation test	1. Control (N=17)<br>2. *cac*^{RNAi} (N=18)	0.010	-0.849	*
	eEPSC jitter – 60Hz	Two sample t-test	1. Control (N=17)<br>2. *cac*^{RNAi} (N=18)	<0.001	-1.452	***
Figure 3G	eEPSC amplitude (pA)	Permutation test	1. Control (N=20)<br>2. *cac*^{RNAi} (N=19)	0.016	0.751	*
Figure 3H	Paired-pulse ratio (10ms interval)	Permutation test	1. Control (N=20)<br>2. *cac*^{RNAi} (N=19)	0.025	-0.731	*
Figure 3I	eEPSC amplitude (pA)	Permutation test	1. Control (N=17)<br>2. *cac*^{RNAi} (N=22)	0.927	-0.028	n.s
Figure 3J	Paired-pulse ratio (10ms interval)	Permutation test	1. Control (N=20)<br>2. *cac*^{RNAi} (N=22)	<0.001	-0.933	***
Figure 4D	J-index probability *cac*^{RNAi} all odors pooled	Permutation test	1. Control (N=10782)<br>2. *cac*^{RNAi} (N=7598)	<0.001	-0.350	***
Figure 4G	J-index probability *cac*^{RNAi} all odors pooled	Permutation test	1. Control (N=5463)<br>2. *cac*^{RNAi} (N=3552)	<0.001	-0.533	***
Figure 5B	Mini frequency (Hz)	Permutation test	1. Control (N=6)<br>2. *cac*^{RNAi} (N=9)	0.984	0.012	n.s
	Mini amplitude (pA)	Two sample t-test	1. Control (N=6)<br>2. *cac*^{RNAi} (N=9)	0.872	0.081	n.s

Figure 5C	Nicotine response (pA)	Permutation test	1. Control (N=23)<br>2. *cac*^{RNAi} (N=22)	0.002	-0.931	**
Figure 5D	# of Brp punctae	Two sample t-test	1. Control (N=7)<br>2. *cac*^{RNAi} (N=10)	0.020	-1.215	*
	Mean intensity of Brp punctae	Two sample t-test	1. Control (N=7)<br>2. *cac*^{RNAi} (N=10)	0.009	-1.401	**
	Mean size of Brp punctae	Two sample t-test	1. Control (N=7)<br>2. *cac*^{RNAi} (N=10)	0.20	0.627	n.s
Figure 6D	Paired pulse ratio-day 0 (10ms)	Two sample t-test	1. Control (N=22)<br>2. *cac*^{RNAi} (N=28)	0.002	-0.915	**
	Paired pulse ratio-day 2-4 (10ms)	Two sample t-test	1. Control (N=13)<br>2. *cac*^{RNAi} (N=15)	0.025	-0.870	*
Figure 7B	Firing rate-control- isopentyl acetate	Permutation test	1. Before GABA blockers (N=45)<br>2. After GABA blockers (N=45)	<0.001	-0.430	***
	Firing rate- *cac*^{RNAi} - isopentyl acetate	Permutation test	1. Before GABA blockers (N=46)<br>2. After GABA blockers (N=46)	0.056	0.144	n.s
	Firing rate-control- isobutyl acetate	Permutation test	1. Before GABA blockers (N=43)<br>2. After GABA blockers (N=43)	<0.001	-0.731	***
	Firing rate- *cac*^{RNAi} - isobutyl acetate	Permutation test	1. Before GABA blockers (N=44)<br>2. After GABA blockers (N=44)	0.842	-0.013	n.s
Figure 7C	Mean variance-control	Permutation test	1. Before GABA blockers (N=88)<br>2. After GABA blockers (N=88)	<0.001	-0.583	***
	Mean variance- *cac*^{RNAi}	Permutation test	1. Before GABA blockers (N=90)<br>2. After GABA blockers (N=90)	0.151	0.088	n.s
	Integration window-control	Permutation test	1. Before GABA blockers (N=88)<br>2. After GABA blockers (N=88)	0.895	0.012	n.s

	Integration window- *cac*^{RNAi}	Permutation test	1. Before GABA blockers (N=90)<br>2. After GABA blockers (N=90)	0.041	0.216	*
Figure 8B	Learning index	One-way ANOVA	1. *Orco-GAL4* (N=40)<br>2. *UAS-cac*^{RNAi} (N=37)<br>3. *Orco-GAL4;UAS-cac*^{RNAi} (N=54)	0.196	0.025	n.s
Figure 8D	p(correct choice)	Permutation test	1. *Orco-GAL4* (N=39)<br>2. *UAS-cac*^{RNAi} (N=40)<br>3. *Orco-GAL4;UAS-cac*^{RNAi} (N=36)	<0.001	0.564	***
Figure 8E	Learning index	One-way ANOVA	1. *Orco-GAL4* (N=43)<br>2. *UAS-cac*^{RNAi} (N=34)<br>3. *Orco-GAL4;UAS- cac*^{RNAi} (N=45)	0.892	0.001	n.s
Figure 8F	p(correct choice)	Permutation test	1. *Orco-GAL4* (N=16)<br>2. *UAS- cac*^{RNAi} (N=16)<br>3. *Orco-GAL4;UAS- cac*^{RNAi} (N=19)	0.559	0.026	n.s
Figure 8G	Learning index	Permutation test	1. *Orco-GAL4* (N=40)<br>2. *UAS- cac*^{RNAi} (N=47)<br>3. *Orco-GAL4;UAS- cac*^{RNAi} (N=34)	0.962	0.0005	n.s
Figure 8H	p(correct choice)	Permutation test	1. *Orco-GAL4* (N=27)<br>2. *UAS- cac*^{RNAi} (N=27)<br>3. *Orco-GAL4;UAS- cac*^{RNAi} (N=29)	<0.001	0.580	***
Figure S2A	Peak ΔF/F isopentyl acetate	Permutation test	1. Control (N=6)<br>2. *cac*^{RNAi} (N=8)	<0.001	2.374	***
	Peak ΔF/F isobutyl acetate	Permutation test	1. Control (N=6)<br>2. *cac*^{RNAi} (N=8)	<0.001	2.956	***
Figure S2B	# of Cac punctae	Two sample t-test	1. Control (N=7)	0.016	1.393	*

			2. *cac*^{RNAi} (N=7)			
Figure S2C	Spikes/sec linalool	Permutation test	1. Control (N=58)<br>2. *cac*^{RNAi} (N=49)	0.014	0.475	*****
	Spikes/sec isopentyl acetate	Permutation test	1. Control (N=71)<br>2. *cac*^{RNAi} (N=49)	<0.001	0.776	*******
	Spikes/sec 2-heptanone	Permutation test	1. Control (N=62)<br>2. *cac*^{RNAi} (N=49)	0.326	0.190	n.s
	Spikes/sec ethyl acetate	Permutation test	1. Control (N=49)<br>2. *cac*^{RNAi} (N=49)	0.291	0.210	n.s
	Spikes/sec isobutyl acetate	Permutation test	1. Control (N=49)<br>2. *cac*^{RNAi} (N=49)	0.007	0.545	******
Figure S6	Time of first spike (ms)	Permutation test	1. Control (N=47)<br>2. *cac*^{RNAi} (N=49)	0.776	0.057	n.s
	STD (ms)	Permutation test	1. Control (N=47)<br>2. *cac*^{RNAi} (N=49)	0.108	-0.329	n.s
Figure S8B	Spikes/sec linalool	Permutation test	1. Control (N=59)<br>2. *syn*^{RNAi} (N=50)	0.04	0.396	*****
	Spikes/sec isopentyl acetate	Permutation test	1. Control (N=72)<br>2. *syn*^{RNAi} (N=49)	<0.001	0.828	*******
	Spikes/sec 2-heptanone	Permutation test	1. Control (N=63)<br>2. *syn*^{RNAi} (N=49)	0.111	0.306	n.s
	Spikes/sec ethyl acetate	Permutation test	1. Control (N=50)<br>2. *syn*^{RNAi} (N=50)	0.026	0.446	*****
	Spikes/sec isobutyl acetate	Permutation test	1. Control (N=50)<br>2. *syn*^{RNAi} (N=50)	0.002	0.619	******
Figure S8D	STD (ms)	Permutation test	1. Control (N=134)<br>2. *syn*^{RNAi} (N=132)	<0.001	-0.851	***

Figure S9	Mean correlation (*wt*)	Permutation test	1. Train 1 (N=945)<br>2. Train 2 (N=766)<br>3. Train 3 (N=714)	<0.001	0.005	***
	Mean correlation (*wt*)- multiple comparisons	Tukey-Kramer	1. Train 1 (N=945)<br>2. Train 2 (N=766)	0.001		**
	Mean correlation (*wt*)- multiple comparisons	Tukey-Kramer	1. Train 1 (N=945)<br>2. Train 3 (N=714)	0.02		*
	Mean correlation (*wt*)- multiple comparisons	Tukey-Kramer	1. Train 2 (N=766)<br>2. Train 3 (N=714)	0.725		n.s
	Mean correlation (cac^{RNAi})	Permutation test	1. Train 1 (N=899)<br>2. Train 2 (N=809)<br>3. Train 3 (N=761)	<0.001	0.014	***
	Mean correlation (cac^{RNAi})- multiple comparisons	Tukey-Kramer	1. Train 1 (N=899)<br>2. Train 2 (N=809)	<0.001		***
	Mean correlation (cac^{RNAi})- multiple comparisons	Tukey-Kramer	1. Train 1 (N=899)<br>2. Train 3 (N=761)	<0.001		***
	Mean correlation (cac^{RNAi})- multiple comparisons	Tukey-Kramer	1. Train 2 (N=809)<br>2. Train 3 (N=761)	0.624		n.s
Figure S10	Peak ΔF/F isopentyl acetate	Two sample t-test	1. Control (N=11)<br>2. cac^{RNAi} (N=12)	<0.001	3.094	***
	Peak ΔF/F isobutyl acetate	Permutation test	1. Control (N=11)<br>2. cac^{RNAi} (N=12)	<0.001	3.516	***
Figure S11	# of Brp punctae	Two sample t-test	1. Control (N=4)<br>2. cac^{RNAi} (N=4)	0.054	1.468	n.s
	Mean intensity of Brp punctae	Permutation test	1. Control (N=4)<br>2. cac^{RNAi} (N=4)	<0.001	-2.206	***

	Mean size of Brp punctae	Two sample t-test	1. Control (N=4)<br>2. *cac*^{RNAi} (N=4)	0.007	-2.431	**
Figure S12	Optimal integration window (ms)	Permutation test	1. Control (N=5234)<br>2. *gluClαRNAi*^{RNAi} (N=3950)	<0.001	-0.230	***
Figure S13A	Learning index	One-way ANOVA	1. *Orco-GAL4* (N=40)<br>2. *UAS-syn*^{RNAi} (N=50)<br>3. *Orco-GAL4;UAS-syn*^{RNAi} (N=54)	0.205	0.022	n.s
Figure S13B	p(correct choice)	Permutation test	1. *Orco-GAL4* (N=26)<br>2. *UAS-syn*^{RNAi} (N=20)<br>3. *Orco-GAL4;UAS-syn*^{RNAi} (N=25)	<0.001	0.3	***

Supplementary References

1. Chang, J. C., Hazelett, D. J., Stewart, J. A. & Morton, D. B. Motor neuron expression of the voltage-gated calcium channel cacophony restores locomotion defects in a Drosophila, TDP-43 loss of function model of ALS. *Brain Res.* **1584**, 39–51 (2014).
