# Supplementary material for: Homeostatic synaptic plasticity rescues neural coding reliability
Source: Nat Commun. 2023 May 24;14:2993. doi: 10.1038/s41467-023-38575-6 (PMC10209050; doi:10.1038/s41467-023-38575-6)
Supplement: Supplementary file 3 — Reporting Summary [file 41467_2023_38575_MOESM3_ESM.pdf]

nature portfolio

Corresponding author(s): Moshe Parnas and Robert J. Kittel

Last updated by author(s): Apr 13, 2023

Reporting Summary

Nature Portfolio wishes to improve the reproducibility of the work that we publish. This form provides structure for consistency and transparency in reporting. For further information on Nature Portfolio policies, see our Editorial Policies and the Editorial Policy Checklist.

Statistics

For all statistical analyses, confirm that the following items are present in the figure legend, table legend, main text, or Methods section.

n/a	Confirmed	
☐	☒	The exact sample size (*n*) for each experimental group/condition, given as a discrete number and unit of measurement
☐	☒	A statement on whether measurements were taken from distinct samples or whether the same sample was measured repeatedly
☐	☒	The statistical test(s) used AND whether they are one- or two-sided<br>*Only common tests should be described solely by name; describe more complex techniques in the Methods section.*
☒	☐	A description of all covariates tested
☐	☒	A description of any assumptions or corrections, such as tests of normality and adjustment for multiple comparisons
☐	☒	A full description of the statistical parameters including central tendency (e.g. means) or other basic estimates (e.g. regression coefficient) AND variation (e.g. standard deviation) or associated estimates of uncertainty (e.g. confidence intervals)
☐	☒	For null hypothesis testing, the test statistic (e.g. *F*, *t*, *r*) with confidence intervals, effect sizes, degrees of freedom and *P* value noted<br>*Give P values as exact values whenever suitable.*
☒	☐	For Bayesian analysis, information on the choice of priors and Markov chain Monte Carlo settings
☒	☐	For hierarchical and complex designs, identification of the appropriate level for tests and full reporting of outcomes
☐	☒	Estimates of effect sizes (e.g. Cohen's *d*, Pearson's *r*), indicating how they were calculated

Our web collection on statistics for biologists contains articles on many of the points above.

Software and code

Policy information about availability of computer code

Data collection	Electrophysiological recordings were performed using pClamp 10.7.
Data analysis	Electrophysiological recordings were analyzed using custom MATLAB code. All statistical testing and parameter extraction were done using custom MATLAB code and PRISM 9.3.0. Figures were prepared using UnivaScatter MATLAB ToolBox, shadedErrorBar function, and custom MATLAB codes written in MATLAB 2018a.<br>Image analysis was done using ImageJ 1.5 3C (National Institutes of Health, Bethesda).<br>The code used to generate the figures are available upon publication in the following GitHub page: https://github.com/ParnasLab/Rozenfeld-et-al-2023.

For manuscripts utilizing custom algorithms or software that are central to the research but not yet described in published literature, software must be made available to editors and reviewers. We strongly encourage code deposition in a community repository (e.g. GitHub). See the Nature Portfolio guidelines for submitting code & software for further information.

Data

Policy information about availability of data

All manuscripts must include a data availability statement. This statement should provide the following information, where applicable:

- Accession codes, unique identifiers, or web links for publicly available datasets
- A description of any restrictions on data availability
- For clinical datasets or third party data, please ensure that the statement adheres to our policy

The data used to generate the figures are available upon publication in the following GitHub page: https://github.com/ParnasLab/Rozenfeld-et-al-2023

Human research participants

Policy information about studies involving human research participants and Sex and Gender in Research.

Reporting on sex and gender	Not relevant
Population characteristics	Not relevant
Recruitment	Not relevant
Ethics oversight	Not relevant

Note that full information on the approval of the study protocol must also be provided in the manuscript.

Field-specific reporting

Please select the one below that is the best fit for your research. If you are not sure, read the appropriate sections before making your selection.

☒ Life sciences ☐ Behavioural & social sciences ☐ Ecological, evolutionary & environmental sciences

For a reference copy of the document with all sections, see nature.com/documents/nr-reporting-summary-flat.pdf

Life sciences study design

All studies must disclose on these points even when the disclosure is negative.

Sample size	Sample sizes are usually larger than the customary in the field. See for example Claridge-Chang et al. Cell 2009 for behavior and Nagel et al. Nat. Neuroscience 2015 for electrophysiology.
Data exclusions	No data were excluded from the analysis.
Replication	Both behavioral and electrophysiological results are obtained from a single fly or a single cell. In each case the results are obtained from an average of a number of repetitions (~50). Thus, each fly or cell is a replication of the experiment.
Randomization	Assignment to experimental groups is according to genotype. Flies of the same genotype were collected into a common vial, from which they were randomly selected for an experiment. In each experiment both experimental and control groups were tested in an alternating manner to control for different confounding variables.
Blinding	Investigators were not blinded to group allocation during data collection and analysis as behavioral and electrophysiolgy analysis was objective. All behavior experiments are performed in an automated manner. For both behavior and electrophysiological recordings all analysis is performed in an automated manner. Therefore, the experimental knowledge of the experimental group has no effect on the results.

Reporting for specific materials, systems and methods

We require information from authors about some types of materials, experimental systems and methods used in many studies. Here, indicate whether each material, system or method listed is relevant to your study. If you are not sure if a list item applies to your research, read the appropriate section before selecting a response.

Materials & experimental systems

n/a	Involved in the study
☐	☒ Antibodies
☒	☐ Eukaryotic cell lines
☒	☐ Palaeontology and archaeology
☐	☒ Animals and other organisms
☒	☐ Clinical data
☒	☐ Dual use research of concern

Methods

n/a	Involved in the study
☒	☐ ChIP-seq
☒	☐ Flow cytometry
☒	☐ MRI-based neuroimaging

Antibodies

Antibodies used

Primary:

- mouse-anti Brp, nc82, gift from Erich Buchner, RRID: AB_2314866
Host organism: mouse
Clonality: monoclonal
Target(s): Bruchpilot, Bruchpilot epitope: C-terminal amino acids 1227-1740 drosophila, mosquito

Proper citation: DSHB Cat# nc82, RRID:AB_2314866)

- rabbit-anti-cacophony, gift from David Morton (Chang et al., 2014, Brain Research)
Host organism: rabbit
Clonality: poyclonal
Target(s): 18 C-terminal amino acids of cacophony (SSRERERDRERLRDRDRD)
not commercially available.

- biotinylated goat anti rabbit IgG, RRID: AB_2337959 [Biotin-SP(long spacer)-AffiniPure Goat Anti-Rabbit IgG (H+L) antibody]
Clonality Polyclonal
Target(s): Rabbit IgG (H+L

Proper citation: Jackson ImmunoResearch Labs Cat# 111-065-003, RRID:AB_2337959)
Vendor: Jackson ImmunoResearch

Secondary:

- goat-anti-mouse STAR RED, RRID: AB_2810982
Host Organism: goat
Clonality: unknown
Target(s): IgG

Proper citation: (Abberior Cat# 2-0002-011-2, RRID:AB_2810982)
Vendor: Abberior

- STAR RED streptavidin, STRED-0120-1MG
Vendor: Abberior

Validation

Primary:

- mouse-anti Brp, nc82, gift from Erich Buchner, RRID: AB_2314866
verified in DSHB Cat# nc82, RRID:AB_2314866)

- rabbit-anti-cacophony, gift from David Morton
verified in Chang et al., 2014, Brain Research

- biotinylated goat anti rabbit IgG, RRID: AB_2337959
verified in Jackson ImmunoResearch Labs Cat# 111-065-003, RRID:AB_2337959)

Animals and other research organisms

Policy information about studies involving animals; ARRIVE guidelines recommended for reporting animal research, and Sex and Gender in Research

Laboratory animals	Transgenic Drosophila melanogaster laboratory strains were used in the study. Both male and female adult flies 0-4 day old). The following transgenes were used: UAS-cacRNAi 85 (VDRC ID 5551), UAS-synRNAi (BDSC_82983), UAS-gluClαRNAi (BDSC_53356), UAS-GABA-B-R2RNAi (BDSC_50608), GH146-QF,QUAS-mCD8-GFP (BDSC_30038), Orco-GAL4 (BDSC_26818), UAS-GCaMP6f (BDSC_52869), 449-QF63, QUAS-mCD8-GFP (BDSC_30002).
Wild animals	The study did not involve wild animals.
Reporting on sex	Study results were obtained from both males and females.
Field-collected samples	The study did not involve samples collected from the field.
Ethics oversight	No ethical approval was required for work involving Drosophila melanogaster.

Note that full information on the approval of the study protocol must also be provided in the manuscript.
